# Cross-sectional pilot study to assess primary healthcare workers’ knowledge of nonalcoholic fatty liver disease in a marginalized community in Mexico

**DOI:** 10.1038/s41598-021-91199-y

**Published:** 2021-06-08

**Authors:** Paulina Vidal-Cevallos, Ana L. Ordóñez-Vázquez, Omar Procopio-Mosso, Rafael Cardoso-Arias, Misael Uribe, Norberto C. Chávez-Tapia

**Affiliations:** 1 Gastroenterology and Obesity Clinic, Medica Sur Clinic and Foundation, C.P. 14050 Mexico City, Mexico; 2 Internal Medicine Department, Medica Sur Clinic and Foundation, C.P. 14050 Mexico City, Mexico; 3 Coordinator of Health Services, Sanitary Jurisdiction 04 Montaña, C.P. 41304 Tlapa de Comonfort, Guerrero Mexico; 4 Medicine and Social Assistance, A.C., C.P. 41304 Tlapa de Comonfort, Guerrero Mexico

**Keywords:** Health care, Gastroenterology, Hepatology

## Abstract

The registered incidence of nonalcoholic fatty liver disease (NAFLD) in primary healthcare centers is lower than expected, suggesting a lack of awareness by primary care healthcare professionals. The implementation of educational tools for healthcare workers has been found to increase timely referral and treatment of patients. We aimed to determine healthcare workers’ knowledge of NAFLD to identify their educational needs in one marginalized region. We performed a cross-sectional survey of 261 healthcare professionals in Tlapa de Comonfort, Guerrero, Mexico from October 2019 to December 2019. We created a questionnaire that assessed domains most relevant to NAFLD knowledge. Two hundred and forty-six questionnaires were completed. Of the respondents, 38.3% were nurses and 63.4% were women. Most nurses identified NAFLD as a prevalent (89%) and preventable (93%) disease. Hypertension (33%) and obesity (84%) were recognized as risk factors. The associations between NAFLD and cancer, cirrhosis and cardiovascular disease were identified by 53%, 67% and 72% of respondents, respectively. The largest gaps were found in diagnostic workup, therapeutic approach and the current treatments. We identify modifiable knowledge gaps in NAFLD. Educational strategies for primary care workers could enhance the identification of patients with NAFLD and prevent complications.

## Introduction

Nonalcoholic fatty liver disease (NAFLD) is a chronic disease that affects approximately 25% of the adult population worldwide. The highest prevalence is reported in South America (30.45%) and the Middle East (31.79%)^1^. In a study conducted in the United States, Hispanics of Mexican origin had a higher prevalence of NAFLD (33%) than Hispanics of other origins (Puerto Rican 18% and Dominican 16%, *P* < 0.01)^2^. The prevalence of NAFLD in Mexico has been predicted to be 29%^3^.

NAFLD is associated with an increased risk of the development of liver fibrosis, cirrhosis and hepatocellular carcinoma^4^. The reported prevalence in four European countries (UK, Spain, Italy and The Netherlands) primary healthcare centers was lower (1.85%, 95% Confidence Interval [CI]: 0.91–2.79) than expected based on its prevalence of 24% (95% CI: 16%–34%), suggesting underdiagnosis and underrecording^5^.

A study conducted by Polanco-Briceno et al.^6^ with 152 primary care physicians demonstrated that up to 50% of the physicians were not familiar with the term nonalcoholic fatty liver disease, or the difference between steatosis and steatohepatitis, even though 58% of the physicians said they were attending patients with the diagnosis of NAFLD.

Another study^7^ found that while 91% of primary healthcare workers recognize the relationship between metabolic syndrome and NAFLD, only 46% claim to have conducted an evaluation for NAFLD in high-risk patients. Weight loss and calorie restriction were considered by 58% as a treatment for NAFLD, but only 27% referred patients for further evaluation by a specialist. The chief impediment for optimizing care was an insufficient understanding of the disease.

On the other hand, the training of nursing staff through the implementation of educational tools has been found to increase detection, treatment and timely referral compared with a single intervention by general practitioners^8^.

The aim of this study was to determine the level of knowledge of NAFLD among primary care healthcare professionals to identify educational needs in a marginalized region in Mexico.

## Results

During the recruitment period, 261 surveys were distributed of which 246 were completed by participants and analyzed. Among the respondents, 100 (40.7%) were nurses, 43 (17.5%) were nursing trainees, 80 (32.5%) were physicians, and 23 (9.3%) were other healthcare workers. The mean age of participants was 28.5 years ± 7.65, 156 (63.4%) were women and 182 (73.9%) worked in community hospitals (Table 1).

**Table 1:** Demographic and workplace characteristics.

	Nurses	Nursing trainees	Physicians	Others^a^
n (%)	100 (40.7%)	43 (17.5%)	80 (32.5%)	23 (9.3%)
Age (years), mean ± SD	28.3 ± 6.9	19.6 ± 1.3	30 ± 6.4	33.2 ± 7.2
Women no. (%)	71 (71%)	33 (76.7%)	47 (58.7%)	5 (21.7%)
Working experience (years), mean ± SD	6.3 ± 6	0.16 ± 0.5	5.8 ± 5	5.8 ± 6.7
**Workplace**				
Community hospital no. (%)	90 (90%)	1 (2.3%)	69 (86.2%)	22 (95.6%)
University no. (%)	2 (2%)	19 (44.9%)	8 (10%)	0
Other^b^ no. (%)	2 (2%)	4 (9.3%)	0	0
Not specified no. (%)	6 (6%)	19 (44.9%)	3 (3.7%)	1 (4.3%)

Others^a^: Health promoters, dentists and psychologists. Others^b^: Mobile Health Units, currently unemployed.

**Figure 1:**
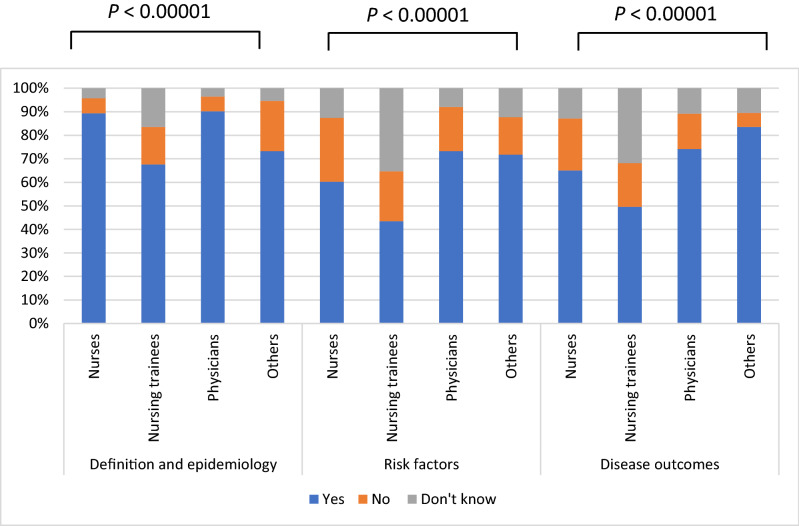
Self-assessment by areas of knowledge. Data are presented divided into three categories: general knowledge (questions 1–4 and 14), knowledge about risk factors (questions 5–10) and knowledge about disease outcomes (questions 11–13) (see Table 2 for the questions).

Definitions and epidemiology of NAFLD were explored through questions 1 to 4 and question 14 (Fig. 1). Eighty-six (86%) of the nurses and 77 (96%) of the physicians reported having heard about NAFLD before, as opposed to 43 (53.6%) and 4 (17.3%) of the nursing trainees and other healthcare workers, respectively. Most of the population considered NAFLD to be a serious but preventable disease (Table 2).

**Table 2:** Results from the “topics related to NAFLD,” “risk factors” and “disease outcomes” questions.

no. (%)	Nurses (n = 100)	Nursing trainees (n = 43)	Physicians (n = 80)	Others (n = 23)
**Definition and epidemiology**				
**Question 1. Have you ever heard of nonalcoholic fatty liver disease?**				
Yes	86 (86%)	23 (53.4%)	77 (96%)	4 (17.3%)
**Question 2. Is nonalcoholic fatty liver disease frequent in your country?**				
Yes	89 (89%)	29 (67.4%)	74 (92.5%)	20 (86.9%)
**Question 3. Is nonalcoholic fatty liver disease a serious illness?**				
Yes	85 (85%)	41 (95.3%)	64 (80%)	23 (100%)
**Question 4. Is nonalcoholic fatty liver disease preventable?**				
Yes	93 (93%)	33 (76.7%)	76 (95%)	23 (100%)
**Question 14. Do you know what metabolic syndrome is?**				
Yes	83 (83%)	18 (9.3%)	69 (86.2%)	12 (52.1%)
**Risk factors for nonalcoholic fatty liver disease**				
**Question 5. Hypertension**				
Yes	33 (33%)	12 (27.9%)	33 (41.2%)	13 (56.5%)
**Question 6. Diabetes Mellitus**				
Yes	55 (55%)	22 (51.6%)	55 (68.7%)	15 (65.2%)
**Question 7. Dyslipidemia**				
Yes	72 (72%)	5 (11.6%)	68 (85%)	16 (69.5%)
**Question 8. Obesity**				
Yes	84 (84%)	22 (51.6%)	73 (91.2%)	20 (86.9%)
**Question 9. Alcohol consumption**				
Yes	64 (64%)	25 (58.1%)	69 (86.2%)	20 (86.9%)
**Question 10. Drug consumption**				
Yes	46 (46%)	25 (58.1%)	52 (65%)	15 (65.2%)
**Disease outcomes**				
**Question 11. Risk factor for cancer**				
Yes	53 (53%)	20 (46.5%)	48 (60%)	15 (65.2%)
**Question 12. Risk factor for cirrhosis**				
Yes	67 (67%)	21 (48.8%)	61 (76.2%)	20 (86.9%)
**Question 13. Risk factor for cardiovascular diseases**				
Yes	72 (72%)	23 (53.4%)	69 (86.2%)	21 (86.9%)

Metabolic syndrome features and alcohol consumption were recognized by 59% of nurses, 43% of nursing trainees, 73% of physicians, and 72% of other healthcare workers as risk factors for the development of NAFLD, with hypertension being the least recognized risk factor, and obesity the most widely recognized risk factor (Table 2). Even so, few among the nurse practitioners and the other healthcare workers knew the definition of metabolic syndrome (Table 2).

Regarding disease outcomes, most of the physicians and other healthcare workers identified the association between NAFLD and cancer (60% and 65.2%, respectively), cirrhosis (76.2% and 86.9%, respectively) and cardiovascular disease (86.2% and 86.9%, respectively). Among nurses and nursing trainees, cardiovascular disease was the most broadly recognized association (72% and 53.4%, respectively) (Table 2).

**Figure 2:**
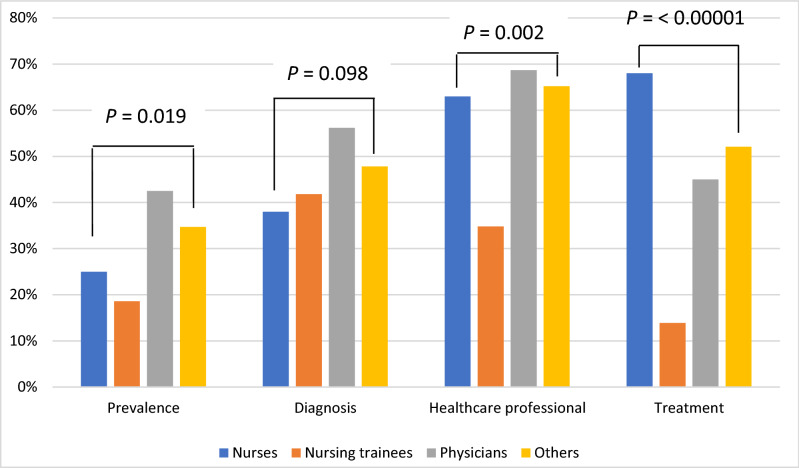
Knowledge about diagnosis and treatment. This area was explored through questions 15–18 (see Table 3 for the questions).

Only 25 (25%) nurses and 34 (42.5%) physicians identified NAFLD as the most frequent liver disease globally. The correct diagnostic methods were identified by 38 (38%) nurses and 45 (56.2%) physicians. Ultrasound was identified as a diagnostic method by 37% of participants, abnormal liver function tests by 30% and liver biopsy by 23% (Fig. 2 and Table 3).

**Table 3:** Results from the “knowledge about diagnosis and treatment” questions.

no. (%)	Nurses (n = 100)	Nursing trainees (n = 43)	Physicians (n = 80)	Others (n = 23)
**Question 15. Which of the following is the most frequent liver disease globally?**				
Correct answer: c. Nonalcoholic fatty liver disease	25 (25%)	8 (18.6%)	34 (42.5%)	8 (34.7%)
**Question 16. Which of the following is a diagnostic test for nonalcoholic fatty liver disease?**				
Correct answers: a. Abdominal CT scan b. Abdominal ultrasound c. Liver biopsy	38 (38%)	18 (41.8%)	45 (56.2%)	11 (47.8%)
**Question 17. Which healthcare professional should evaluate a patient for suspected nonalcoholic fatty liver disease?**				
Correct answers: a. Gastroenterologist b. Nutritionist	63 (63%)	15 (34.8%)	55 (68.7%)	15 (65.2%)
**Question 18. Which of the following do you consider to be a treatment for nonalcoholic fatty liver disease?**				
Correct answers: a. Weight loss b. Surgery d. Vitamin E	68 (68%)	6 (13.9%)	36 (45%)	12 (52.1%)

Sixty-three (63%) nurses and 55 (68.7%) physicians responded that gastroenterologists and/or nutritionists should evaluate a patient for suspected NAFLD. Weight loss, surgery and vitamin E were poorly recognized as currently approved treatments for NAFLD by only 68 (68%) nurses and 36 (45%) physicians (Fig. 2 and Table 3).

## Discussion

Our study aimed to assess knowledge about NAFLD in healthcare professionals working in a marginalized region in Mexico to identify educational needs. Four domains were evaluated for that purpose: definitions and epidemiology, risk factors, disease outcomes and diagnosis, and treatment strategies.

We found that 92.5% of the physicians considered NAFLD to be a prevalent disease. However, only 42.5% identified it as the most prevalent liver disease globally. This finding agrees with that reported in a study in Wisconsin, USA where 83% of primary care physicians reported NAFLD as an important health problem even though 85% underestimated its prevalence^7^. Another US-based study, which included physicians and nurse practitioners reported that 31% underestimated the prevalence of NAFLD^9^. The lack of awareness of NAFLD, combined with the fact that it is largely an asymptomatic disease, is an important issue that may contribute to affected individuals remaining undiagnosed and untreated.

In our study, alcohol consumption was the most widely identified risk factor, which is in agreement with a study in Queensland, Australia where primary care physicians considered alcohol consumption to be strongly associated with NAFLD^10^. Because the diagnosis of NAFLD requires the exclusion of alcohol consumption as a secondary cause, the fact that it was considered a risk factor reflects a misconception in the definition.

On the subject of metabolic syndrome, 57.6% of respondents in our study reported having some knowledge about this condition, with obesity being the best-associated risk factor and hypertension the least associated. Other studies have had similar results. In one study in the United States, only 13% of respondents could associate NAFLD with metabolic syndrome^6^. Another US study^9^ found that even though 75% of primary care physicians screened people with metabolic syndrome for NAFLD, only 24% screened people with hypertension. These findings suggest that although most primary care physicians have some notion of metabolic syndrome, they do not understand its complexities sufficiently well enough to identify its components separately as risk factors for NAFLD.

Concerning disease outcomes, the study participants could identify the risk for developing cardiovascular diseases, cirrhosis and cancer. These data are similar to the findings of a study that included general practitioners and nurses, where it was identified that 65.7% of the participants associated simple steatosis with an increased incidence of cardiovascular disease, 61.8% with increased liver-related mortality and 47.1% with a higher risk of cirrhosis^10^.

With regard to diagnostic workup, only 45.9% of the participants were able to indicate the correct diagnostic strategy. This finding contrasts with what was found in another study, where abnormal liver enzymes (92%) and abdominal ultrasound (87.3%) were the tests of choice for the assessment of NAFLD^10^. In relation to diagnostic method, these results reflect a lack of awareness in our population that is greater than that reported in the literature.

On the topic of assessment of people with NAFLD by a specialist, 57.9% correctly identified the need for a nutritionist and a gastroenterologist for the evaluation of these patients. Other studies have found that 28–70.6% of general practitioners considered referral to a gastroenterologist or a hepatologist. In these studies, the primary reasons for referral were an increase or persistent alteration in levels of circulating transaminases, participation in clinical trials or monitoring for liver-related complications^6, 9^.

The weakest domain for the study subjects was treatment. Currently approved treatments for NAFLD were recognized correctly by only 44.75% of the participants. Alarmingly, only 45% of primary care physicians had knowledge of NAFLD treatment alternatives. Other studies have found that the most broadly utilized treatment option is referral to a dietitian (58.92%), while pharmacological treatment is less frequently used^7^.

A study that included primary care healthcare providers reported that 70% of general practitioners prescribed some treatment for people with suspected NAFLD, but only 47% felt confident treating NAFLD^6^. Surprisingly, only 1.6% of our participants considered there is no cure for NAFLD, in contrast with what has been reported by another study where 40% of providers believed there is no effective treatment for NAFLD^9^.

The main strengths of our study are that we evaluated healthcare providers working in the primary care setting (physicians, nurses, nursing trainees and other healthcare givers) in a marginalized region of a developing nation. To our knowledge, this is the first study conducted on this subject in a region with such characteristics. This is important because Mexico is an underdeveloped country with among the highest reported prevalence both of NAFLD and of metabolic syndrome globally. It is particularly noteworthy that a previous study performed in Tlapa de Comonfort found that the prevalence of undiagnosed cirrhosis was 7.3%, with obesity being the main risk factor (OR 2.42, 95% CI 1.02–6.3, *P* = 0.046)^11^.

Limitations include the use of purposive sampling for data collection because attendees for the conferences could be more motivated toward further education and may potentially overestimate their knowledge. Because the sample was not randomized, this could result in selection bias. An important limitation is that there are no validated instruments to measure NAFLD knowledge in primary healthcare providers. Because this was an exploratory study, a questionnaire was created for this study. An additional limitation is that the questionnaire did not assess knowledge, but rather, measured the self-appraisal of knowledge, which may also lead to overestimation of the results. Another limitation is the restriction of the geographical area. Results could only be compared among areas with similar resource and educational restrictions. Despite these limitations, we expect the average knowledge of NAFLD among other primary healthcare providers from this region to be similar. In this particular municipality, the main causes of mortality are diabetes mellitus and neoplasms^12^. A focus on these conditions may hamper the prioritization of NAFLD, which in turn may be reflected in a lack of awareness of NAFLD in the community.

## Conclusions

Through our survey, we were able to identify modifiable clinical practice gaps in the diagnosis, treatment and prevention of further complications in people with NAFLD. In a setting with resources as limited as this one, identification of these gaps could help improve the training of primary care workers and enhance the identification of high-risk patients to prevent complications and decrease disease burden. Further research is necessary to investigate the influence language barriers, lack of access to basic housing services and educational level have on the understanding of NAFLD in a setting with limited health resources.

## Methods

We conducted a cross-sectional survey about knowledge of NAFLD risk factors, diagnosis and treatment in Tlapa de Comonfort, Guerrero, Mexico. Tlapa de Comonfort is an urbanized city in the mountain region of Guerrero, in southern Mexico. As of 2010, it had 81,419 inhabitants, with a population density of 133.25 people/km^2^. It also has a considerable indigenous population (32% of the population speaks an indigenous language). With respect to economic and social development, 76% of the population live in poverty (32% in extreme poverty without access to food), 35% of the population has educational backwardness, 39.55% do not have access to health services and 63.9% lack access to basic housing services^12^.

From October 2019 to December 2019, we recruited a convenience sample through conferences given by the primary investigators and visits by coinvestigators to a Care Center belonging to a nonprofit organization, 12 Primary Care Centers, 8 Community Hospitals and 28 Mobile Medical Units belonging to the State of Guerrero Health System. The questionnaires were distributed during visits and conferences on topics not related to NAFLD by people not involved in the study and by coinvestigators. Questionnaires were printed and handed out to the respondents upon arrival at the conferences. Completed questionnaires were self-applied and handed back when finished.

Respondents included nurses, nurses in training, physicians, and other healthcare workers (such as social workers, dentists and health promoters). Participants were included if they were 18 years or older, had finished a health-related degree or were enrolled in one, and worked in any of the participating health centers. Participants were excluded if they did not speak Spanish fluently.

We created a questionnaire (Supplementary Material Table S1) that assessed selected key domains considered by the authors most relevant to NAFLD knowledge. Questions were developed after reviewing information in the current literature and current European Association for the Study of the Liver (EASL)^13^ and American Association for the Study of Liver Diseases (AASLD guidelines)^14^. This questionnaire consisted of 4 demographic questions, 14 items exploring topics related to NAFLD that required a “yes,” “no” or “don’t know” response and 4 multiple-choice questions evaluating knowledge on diagnosis and treatment.

Questionnaire data were grouped into four categories for analysis: definitions and epidemiology (questions 1–4, and 14), knowledge about risk factors (questions 5–10), knowledge about disease outcomes (questions 11–13) and questions about diagnosis and treatment (questions 15–18). Multiple-choice questions were deemed correct if no incorrect option was selected and any number of the correct options was selected.

### Statistical analysis

The questionnaire data were inputted onto a database and analyzed using Microsoft Excel for Mac. Frequency and descriptive analyses were completed for all quantitative questions, continuous variables with normal distribution were expressed as mean ± standard deviation (SD) and those with non-normal distribution variables were reported as median (interquartile range). Frequencies for categorical variables are displayed as percentages. The comparison between groups of different healthcare providers was done with the chi-square test.

The present study was approved by the Ethics Committee of the Medica Sur Clinic and Foundation, Mexico City, Mexico, and complies with the basic principles of human research following the Helsinki Declaration of the Medical Association (Helsinki Finland 1964 last amendment at the 52nd General Assembly, in Fortaleza, Brazil, October 2013). Written informed consent was obtained from all participants or if subjects were under 18 years, from a parent and/or legal guardian. The modality of our instrument for evaluation ensures no risk to the anonymity of the participants and the data collected were handled confidentially.

## Supplementary Information

Supplementary Table S1.
