# Supplementary material for: Cross-sectional pilot study to assess primary healthcare workers’ knowledge of nonalcoholic fatty liver disease in a marginalized community in Mexico
Source: Sci Rep. 2021 Jun 8;11:12100. doi: 10.1038/s41598-021-91199-y (PMC8187373; doi:10.1038/s41598-021-91199-y)
Supplement: Supplementary file 1 — Supplementary Table S1. [file 41598_2021_91199_MOESM1_ESM.docx]

**Title**

“Cross-sectional pilot study to assess primary healthcare workers’ knowledge of nonalcoholic fatty liver disease in a marginalized community in Mexico”

**Author names:**

Paulina Vidal-Cevallos

Gastroenterology and Obesity Clinic, Medica Sur Clinic and Foundation.

Puente de Piedra 150, col. Toriello Guerra, C.P. 14050, Mexico City, Mexico.

E-mail: [paulina.vice@gmail.com](mailto:paulina.vice@gmail.com)

Ana L. Ordóñez-Vázquez

Internal Medicine Department, Medica Sur Clinic and Foundation.

Puente de Piedra 150, col. Toriello Guerra, C.P. 14050, Mexico City, Mexico.

E-mail: [annordonez.07@hotmail.com](mailto:annordonez.07@hotmail.com)

Omar Procopio-Mosso

Coordinator of Health Services, Sanitary Jurisdiction 04 Montaña.

Añorve #84, col. San Francisco, C.P. 41304, Tlapa de Comonfort, Guerrero México.

E-mail: [promar2_@hotmail.com](mailto:promar2_@hotmail.com)

Rafael Cardoso-Arias

Medical Director at Medicine and Social Assistance, A.C.

Amapolas 99, col. Aviación, C.P. 41304, Tlapa de Comonfort, Guerrero México.

E-mail: [rcardoso@mas.org.mx](mailto:rcardoso@mas.org.mx)

Misael Uribe

Gastroenterology and Obesity Clinic, Medica Sur Clinic and Foundation.

Puente de Piedra 150, col. Toriello Guerra, C.P. 14050, Mexico City, Mexico.

E-mail: [muribe@medicasur.org.mx](mailto:muribe@medicasur.org.mx)

**Corresponding author:**

Norberto C. Chávez-Tapia

Medica Sur Clinic and Foundation, Mexico City, Mexico.

Puente de Piedra 150, col. Toriello Guerra, C.P. 14050, Mexico City, Mexico.

Telephone: +52 55 54246850

e-mail: [nchavezt@medicasur.org.mx](mailto:nchavezt@medicasur.org.mx)

**SUPPLEMENTARY MATERIAL**

| **SUPPLEMENTARY MATERIAL TABLE S1.** Non-alcoholic fatty liver disease knowledge questionnaire | | | |
| --- | --- | --- | --- |
| **1.** Have you ever heard of non-alcoholic fatty liver disease? | Yes | No | Don’t know |
| **2.** Do you consider non-alcoholic fatty liver disease to be frequent in your country? | Yes | No | Don’t know |
| **3.** Do you consider non-alcoholic fatty liver disease to be a serious illness? | Yes | No | Don’t know |
| **4.** Do you consider non-alcoholic fatty liver disease to be a preventable disease? | Yes | No | Don’t know |
| **5.** Do you reckon that hypertension favours the development of non-alcoholic fatty liver disease? | Yes | No | Don’t know |
| **6.** Do you reckon that Diabetes Mellitus favours the development of non-alcoholic fatty liver disease? | Yes | No | Don’t know |
| **7.** Do you reckon that dyslipidemia favours the development of non-alcoholic fatty liver disease? | Yes | No | Don’t know |
| **8.** Do you reckon that obesity favours the development of non-alcoholic fatty liver disease? | Yes | No | Don’t know |
| **9.** Do you reckon that alcohol consumption favours the development of non-alcoholic fatty liver disease? | Yes | No | Don’t know |
| **10.** Do you consider that there are medications that promote the development of non-alcoholic fatty liver disease? | Yes | No | Don’t know |
| **11.** Do you believe non-alcoholic fatty liver disease to favour the development of cancer? | Yes | No | Don’t know |
| **12.** Do you believe that non-alcoholic fatty liver disease favours the development of cirrhosis? | Yes | No | Don’t know |
| **13.** Do you believe that non-alcoholic fatty liver disease favours the development of cardiovascular diseases (e.g. coronary artery disease, stroke or cerebral haemorrhage)? | Yes | No | Don’t know |
| **14.** Do you know what metabolic syndrome is? | Yes | No | Don’t know |
| **15.** Which of the following is the most frequent liver disease globally? | | | |
| 1. Hepatitis C Virus infection | | | |
| 1. Alcohol consumption | | | |
| 1. Non-alcoholic fatty liver disease | | | |
| 1. Don´t know | | | |
| **16.** Which of the following is a diagnostic test for non-alcoholic fatty liver disease? | | | |
| 1. Abdominal CT scan | | | |
| 1. Liver function tests | | | |
| 1. Abdominal ultrasound | | | |
| 1. Liver biopsy | | | |
| **17.** Which healthcare professional should evaluate a patient for suspected non-alcoholic fatty liver disease? | | | |
| 1. Primary care physician | | | |
| 1. Surgeon | | | |
| 1. Gastroenterologist | | | |
| 1. Nutritionist | | | |
| 1. Gynaecologist | | | |
| **18.** Which of the following do you consider to be the treatment of non-alcoholic fatty liver disease? | | | |
| 1. Weight loss | | | |
| 1. Surgery | | | |
| 1. Supplements | | | |
| 1. Vitamin E | | | |
| 1. Metformin | | | |
| 1. It has no cure | | | |

| **SUPPLEMENTARY MATERIAL TABLE S2.** Non-alcoholic fatty liver disease knowledge questionnaire (Spanish Original) | | | |
| --- | --- | --- | --- |
| **1.** ¿Ha escuchado alguna vez sobre Enfermedad por Hígado Graso? | Sí | No | No lo sé |
| **2.** ¿Considera que es una enfermedad frecuente en nuestro país? | Sí | No | No lo sé |
| **3.** ¿Considera que es una enfermedad grave? | Sí | No | No lo sé |
| **4.** ¿Considera que es una enfermedad prevenible? | Sí | No | No lo sé |
| **5.** ¿Usted cree que la hipertensión arterial favorece el desarrollo de hígado graso? | Sí | No | No lo sé |
| **6.** ¿Usted cree que la Diabetes Mellitus favorece el desarrollo de hígado graso? | Sí | No | No lo sé |
| **7.** ¿Usted cree que la dislipidemia favorece el desarrollo de hígado graso? | Sí | No | No lo sé |
| **8.** ¿Usted cree que la obesidad favorece el desarrollo de hígado graso? | Sí | No | No lo sé |
| **9.** ¿Usted cree que el consumo de alcohol favorece el desarrollo de hígado graso? | Sí | No | No lo sé |
| **10.** ¿Usted cree que existen medicamentos que favorecen el desarrollo de hígado graso? | Sí | No | No lo sé |
| **11.** ¿Usted cree que el hígado graso favorece el desarrollo de cáncer? | Sí | No | No lo sé |
| **12.** ¿Usted cree que el hígado graso favorece el desarrollo de cirrosis? | Sí | No | No lo sé |
| **13.** ¿Usted cree que el hígado graso favorece el desarrollo de enfermedades cardiovasculares (ej. Infarto al corazón, embolia o hemorragia cerebral)? | Sí | No | No lo sé |
| **14.** ¿Sabe qué es el síndrome metabólico? | Sí | No | No lo sé |
| **15.** ¿Cuál de las siguientes es la enfermedad hepática más frecuente en el mundo? | | | |
| 1. Infección por Virus de Hepatitis C | | | |
| 1. Consumo de alcohol | | | |
| 1. Hígado graso no alcohólico | | | |
| 1. No lo sé | | | |
| **16.** ¿Cuál de los siguientes es método diagnóstico para hígado graso? (Puedes seleccionar más de una respuesta) | | | |
| 1. Tomografía de abdomen | | | |
| 1. Pruebas de función hepática | | | |
| 1. Radiografía de abdomen | | | |
| 1. Ultrasonido de abdomen | | | |
| 1. Biopsia hepática | | | |
| **17.** ¿Con qué especialista de la salud enviaría a valoración a un paciente con probable hígado graso? (Puedes seleccionar más de una respuesta) | | | |
| 1. Médico general | | | |
| 1. Cirujano | | | |
| 1. Gastroenterólogo | | | |
| 1. Nutriólogo | | | |
| 1. Ginecólogo | | | |
| **18.** ¿Cuál considera que es el tratamiento del hígado graso? (Puedes seleccionar más de una respuesta) | | | |
| 1. Reducción de peso | | | |
| 1. Cirugía | | | |
| 1. Suplementos alimenticios | | | |
| 1. Vitamina E | | | |
| 1. Metformina | | | |
| 1. No tiene cura | | | |
